# Charge transport in a DNA model with solvent interaction

**DOI:** 10.1007/s10867-018-9503-x

**Published:** 2018-07-03

**Authors:** H. Ngoubi, G. H. Ben-Bolie, T. C. Kofané

**Affiliations:** 1 Laboratory of Biophysics, Department of Physics, Faculty of Science, University of Yaounde I, P.O. Box 812, Yaounde, Cameroon; 2 Laboratory of Nuclear Physics, Department of Physics, Faculty of Science, University of Yaounde I, P.O. Box 812, Yaounde, Cameroon; 3 Laboratory of Mechanics, Department of Physics, Faculty of Science, University of Yaounde I, P.O. Box 812, Yaounde, Cameroon

**Keywords:** Charge transport, Peyrard–Bishop model, Solvent interaction

## Abstract

The charge transport in the modified DNA model is studied by taking into account the factor of solvent and the effect of coupling motions of nucleotides. We report on the presence of the modulational instability (MI) of a plane wave for charge migration in DNA and the generation of soliton-like excitations in DNA nucleotides. By applying the continuum approximation, we show that the original differential-difference equation for the DNA dynamics can be reduced to a set of three coupled nonlinear equations. The linear stability analysis of wave solutions of the coupled systems is performed and the growth rate of instability is found numerically. We also investigate the impact of solvent interaction. The solvent factor introduces a new behavior to the wave patterns, modifying also the intrinsic properties of localized structures. In the numerical simulations, we show that the solitons exists when taking into account the effect of solvent and confirms an highest propagation of localized structures in the systems. The effect of solvent forces introduces a robustness behavior to the formed patterns, reinforcing the idea that the information in the DNA model is confined and concentrated to specific regions for efficiency. We also show that the localized structures can be disappeared with the highest value of solvent factor and thereafter the information within the molecule is not perceptible or not transmitted to another sites.

## Introduction

Charge transfer through biomolecular systems is one of the most promising ongoing investigations in biophysics and nanotechnology. So, understanding the charge-transport mechanisms is essential for the development of molecular electronic devices. In this context, the study of different modes of charge transfer, both theoretical and experimental, is devoted to illumination of the mechanisms of charge transport [1–3]. In this context, Cherstvy et al. [4] studied the non-linear effect in charge transport. Sun-Yong et al. [5] studied the effect of the base pair on the charge transport in double-strand DNA. The authors proved that the charge transport decreases when the base pairs are opened. Dirk et al. [6] proved that the soliton is responsible for the energy transfer and localization. Toko et al. [7] investigated the propagation of localized structures in a DNA model, which takes into account helicity and solvent interaction by using the Peyrard and Bishop model. The authors showed that the solvent interaction term modulates and increases the width of the pulses [7]. In recent years, localized and nonlinear excitations [8–16], (solitons, discrete breathers, intrinsic localized modes) have been drawing increasing attention and are widely believed to be responsible for several effects in molecular chains, such as charge and thermal conductivity, energy transfer, and localization. A particular interesting discrete system that support solitons and localized modes is desoxyribonucleic acid, or DNA. In this system, localization of energy has been suggested as a precursor of the transcription bubble [9], and moving localized oscillations as a method of transport of information along the double strand [12].

In recent years, due to experiments on single molecules of DNA, models with two and more degrees of freedom have been introduced with insistence on the radial and torsional aspects. We take as an example Barbi, Cocco and Peyrard [12], and later improved by Cocco and Monasson [13, 14]. In recent research, localized structure waves are paid too much attention while studying DNA dynamics in the presence of some perturbations. The impact of damping and thermal fluctuations on lattice soliton patterns due to the presence of thermal noise were studied by Arévalo et al. [15, 16] and Ekobena et al. [17], who demonstrated a gradual increase energy soliton pattern due to the presence of thermal noise in the bi-exciton molecular chain. Kalosakas et al. [18] shows that the thermal fluctuations do not destroy completely the soliton localization in biological molecule. Many authors, for example Samora-Sillero et al. [19], have shown that the most standard mechanism through which bright solitons or solitary wave structures appear is through the activation of modulational instability (MI) of plane waves. Fialko and Laklno [20, 21]has studied the transport of charge and hole along the short DNA molecule by using the Peyrard–Bishop–Holstein (PBH) model [22, 23]. These authors investigate the impact of long-range transfer of charges through the DNA molecule.

Then, Kornyshev et al. [24] studied the process of denaturation in a DNA model by nonlinear effects of torsional deformations. Hidayat et al. [25] investigated the impact of the viscosity in the process of denaturation. The result obtained showed how at a certain temperature the increase of the viscosity coefficient will decrease the melting temperature. The way that the solvent factor affects the transport of charges via MI, in the DNA model with the vibrational and rotational coupled motions, is the main focus of the present work. This work is organized as follows. In Section 2, we propose the model and we derive the equations of motion. In Section 3, linear analysis is also studied in this part and predictions for some localized structure formation. The validity of this analysis is proved by numerical simulations in Section 4 where we will point out the effect of solvent interaction, which will lead to a conclusion in Section 5.

## Model and equations of motion

Structural dynamics of DNA base-pair is described by [26].

1 $$\begin{array}{@{}rcl@{}} H_{1}&=&\sum\limits_{j}\left\{\frac{p_{w_{j}}^{2}}{2m}+\frac{p_{\lambda_{j}}^{2}}{2m} +\frac{p_{\phi_{j}}^{2}}{2I}+\frac{p_{\theta_{j}}^{2}}{2I}\right\}+ \sum\limits_{j}\left\{\frac{k}{2}[(w_{j}-w_{j-1})^{2}+(\lambda_{j}-\lambda_{j-1})^{2}]\right\}\\ && + \sum\limits_{j}\left\{\frac{\xi}{2}[(\phi_{j}-\phi_{j-1})^{2}+(\theta_{j}-\theta_{j-1})^{2}]\right\}+\sum\limits_{j} U(y_{j})+\sum\limits_{j} V_{sol}({y_{j}}). \end{array} $$

where

2 $$\begin{array}{@{}rcl@{}} w_{j}&=&\frac{u_{j}+v_{j}}{\sqrt{2}} \\ \lambda_{j}&=&\frac{u_{j}-v_{j}}{\sqrt{2}}, \end{array} $$

The displacement of the *H* bond between two adjacent discs *j*, used in this present work, can be written as [26]

3 $$ y_{j}=(u_{j}-v_{j}+d + 2r)-r(\cos\theta_{j}+\cos\phi_{j}). $$

In the above equation, $ p_{w_{j}} $ (or $ p_{\lambda _{j}} $) and $ p_{\phi _{j}} $ (or $ p_{\theta _{j}} $) are the linear and angular moments, respectively. The parameters *u*_*j*_ and *v*_*j*_ represent the transverse and the longitudinal displacements from equilibrium (stretching) of the base-pair at site *j* and the rotational motions characterized by *𝜃*_*j*_ and *ϕ*_*j*_ [26]. We consider that the mass *m* and the inertia of moment *I* are the same value for all nucleotides, and also the constants *k* and *ξ*. In this present work, we consider only the artificial and the homogeneous DNA molecule. Consequently, all bases pair are identical. The Morse potential is given by [27].

4 $$ U ({y_{j}} ) \,=\, D_{j}\left[ \exp \left( - a(y_{j}-y_{0})\right) \,-\, 1 \right]^{2} \,=\, D_{j}\left[ {\exp \left( {- a\sqrt{2}\lambda_{j}\,-\,g(\phi_{j},\theta_{j}) } \right) \,-\, 1} \right]^{2}. $$

where *D*_*j*_ is the energy of dissociation of the base pair and *a* is the parameter with dimension of inverse length, *y*_0_ = 2*r* + *d* is the equilibrium point (distance between the centers of the discs), and the function *g*(*ϕ*_*j*_, *𝜃*_*j*_) is given by

5 $$ g(\phi_{j},\theta_{j})=r(\cos\phi_{j}+\cos\theta_{j}). $$

Since all biological molecules like DNA are always in contact with a thermal bath, the above extended PB model will be coupled by the solvent interaction term [28–30]:

6 $$ V_{sol}({y_{j}})=D_{j} f_{s}\left( \tanh\left( \frac{\sqrt{2}\lambda_{j}-g(\phi_{j},\theta_{j})}{l_{s}}\right)-1\right), $$

The term resulting from the solvent has the effect of modulating the amplitude of the soliton *f*_*s*_ and by a term *l*_*s*_ setting the range of this potential. All these external factors contribute to increase the energy and the charge. In the following, the above extended PB model will be coupled to the excitons. Thus, the Hamiltonian of excitons for the system can be written as.

7 $$ H_{2} = - \sum\limits_{j} {V\left( {B_{j}^ + B_{j + 1} + B_{j}^ + B_{j - 1} }, \right)} $$

In this Hamiltonian, $B_{j}^{+}$ and *B*_*j*_ are creation and annihilation operators, respectively, for charge carrier at the *jth* base pair of the double strand, and *V* represents the coupling transfer integral between π orbital at adjacent base pairs. The simplest way to take into account the impact of charge–lattice interaction is through a linear coupling of charge’s on-site energy with the displacements *λ*_*j*_, as proposed in Holstein model [22, 23]. So that the Hamiltonian interaction of the system can be expressed as:

8 $$ H_{3} = \sum\limits_{j} {\chi \lambda_{j} } B_{j}^ + B_{j}. $$

where *χ* is the coupling vibrational and rotational constant. The equations of motion are in the Appendix.

## MI analysis and DNA wave patterns

In order to perform the linear stability analysis of system (57)–(59), we assume that:

9 $$\begin{array}{@{}rcl@{}} \varphi=\varphi_{0}\exp i(k x-w_{0}t),\\ \lambda=\lambda_{0} ,\\ \psi=\psi_{0}\exp i(k x-w_{0}t), \end{array} $$

with real constants *w*_0_, *λ*_0_, *ψ*_0_ and *φ*_0_ is a complex constant, *k* and *w*_0_ are wave number and frequency, respectively, of the system without perturbation. Introducing the above relation into (57)–(59), we get:

10 $$ {w_{0}^{2}}-w_{0}+Q^{\prime}= 0. $$

where

11 $$\begin{array}{@{}rcl@{}} Q^{\prime}=-\left( \frac{1}{P_{1}}(Q_{1}+Q_{2} \lambda_{0} \chi)+Q_{3}\lambda_{0}+Q_{4}{\lambda_{0}^{2}}+Q_{5}-{\beta\gamma\lambda_{0}^{3}}\right). \end{array} $$

and

12 $$ k^{4}-\mu k^{2}+\mu_{1}= 0. $$

where

13 $$ \mu=-\frac{1}{{P_{1}^{2}}}(1 + 2P_{1}Q_{1}+ 2P_{1}Q_{2}\chi\lambda_{0}). $$

14 $$ \mu_{1}= \left[- \frac{P_{1} \mu}{2}-\frac{1}{2P_{1}}\right]^{2}+Q_{3}\lambda_{0}+Q_{4}{\lambda_{0}^{2}}+Q_{5}-{\beta\gamma\lambda_{0}^{3}}. $$

Equation (10) has the following solution

15 $$ w_{0+}=\frac{1+\sqrt{1-4Q^{\prime}}}{2}, $$

16 $$ w_{0-}=\frac{1-\sqrt{1-4Q^{\prime}}}{2}, $$

and (12) have fourth solutions giving by:

17 $$ k_{+}^{2}=\frac{\mu+\sqrt{\mu^{2}-4\mu_{1}}}{2}, $$

18 $$ k_{-}^{2}=\frac{\mu-\sqrt{\mu^{2}-4\mu_{1}}}{2}, $$

Adding a small perturbation in above the equilibrium state, that is

19 $$\begin{array}{@{}rcl@{}} \varphi=(\varphi_{0}+\epsilon\varphi_{1})\exp i(K x+w_{0}t),\\ \lambda=\lambda_{0}+\epsilon\lambda_{1},\\ \psi=(\psi_{0}+\epsilon\psi_{1})\exp i(K x+w_{0}t), \end{array} $$

and using it in (57)–(59), with the help of condition (10), we write *φ*_0_ = *a*_1_ + *ia*_2_, *φ*_1_ = *u* + *iv*, *ψ*_0_ = *b*_1_ + *ib*_2_, *ψ*_1_ = *u*_1_ + *iv*_2_ where we are taking *a*_1_ = *a*_2_ for the sake of simplicity, and we separate imaginary and real parts as follows:

20 $$ P_{1}\frac{\partial^{2}u}{\partial x^{2}}-\frac{\partial v}{\partial t}+Q_{1}u+Q_{2}a_{1}\lambda_{1}= 0. $$

21 $$ P_{1}\frac{\partial^{2}v}{\partial x^{2}}+\frac{\partial u}{\partial t}+Q_{1}v+Q_{2}a_{2}\lambda_{1}= 0. $$

22 $$ c_{0}\frac{\partial^{2}\lambda_{1}}{\partial x^{2}}+\frac{\partial^{2} \lambda_{1}}{\partial t^{2}}+(A-2B\lambda_{0}+ 3W_{g}{\lambda_{0}^{2}})\lambda_{1}+ 2c_{2}(ua_{1}+a_{2}v)= 0. $$

23 $$\begin{array}{@{}rcl@{}} && \frac{\partial^{2}u_{1}}{\partial t^{2}}+ 2w_{0}\frac{\partial v_{2}}{\partial t}-{w_{0}^{2}}u_{1}+c_{1}\left( \frac{\partial^{2} u_{1}}{\partial x^{2}}-2K\frac{\partial v_{2}}{\partial x}-K^{2}u_{1}\right)-Q_{3}b_{1}\lambda_{1}-Q_{3}\lambda_{0} u_{1}\\ &&\quad +\, 2Q_{4}\lambda_{0}b_{1}\lambda_{1}-3b_{1}{\lambda_{0}^{2}}\gamma\lambda_{1}-{\beta\gamma\lambda_{0}^{3}}u_{1}+Q_{5}u_{1}= 0. \end{array} $$

24 $$\begin{array}{@{}rcl@{}} &&\frac{\partial^{2}v_{2}}{\partial t^{2}}-2w_{0}\frac{\partial u_{1}}{\partial t}-{w_{0}^{2}}v_{2}+c_{1}\left( \frac{\partial^{2} v_{2}}{\partial x^{2}}+ 2K\frac{\partial u_{1}}{\partial x}-K^{2}v_{2}\right)-Q_{3}b_{2}\lambda_{1}-Q_{3}\lambda_{0} v_{2}\\ &&\quad +\, 2Q_{4}\lambda_{0}b_{2}\lambda_{1}-3b_{2}{\lambda_{0}^{2}}\gamma\lambda_{1}-{\beta\gamma\lambda_{0}^{3}}v_{2}+Q_{5}v_{2}= 0. \end{array} $$

Then, we insert

25 $$ u=u_{01}\exp i(K_{1}x-{\Omega} t)+cc. $$

26 $$ v=v_{01}\exp i(K_{1}x-{\Omega} t)+cc. $$

27 $$ \lambda_{1}=\lambda_{01}\exp i(K_{1} x-{\Omega} t)+cc. $$

28 $$ u_{1}=u_{10}\exp i(K_{1} x-{\Omega} t)+cc. $$

29 $$ v_{2}=v_{20}\exp i(K_{1} x-{\Omega} t)+cc. $$

into (20–24), where *K*_1_ and Ω are the perturbation wave number and frequency, respectively. *cc* is the complex conjugate. We arrive to five coupled linear equations

30 $$ M(u_{01},v_{01},\lambda_{01},u_{10}, v_{20})^{T}= 0. $$

with $M=\left ({\begin {array}{l} m_{11}\\ \\ -i{\Omega }\\ \\ m_{31}\\ \\ 0\\ 0\\ \par \end {array}\begin {array}{l} i{\Omega }\\ \\ m_{11}\\ \\ m_{32}\\ \\ 0\\ 0\\ \par \end {array} \begin {array}{l} m_{13}\\ \\ m_{23}\\ \\ -{\Omega }^{2}+m_{33}\\ \\ m_{43}\\ \\ m_{43}\\ \par \end {array} \begin {array}{l} 0\\ \\ 0\\ \\ 0\\ \\ -{\Omega }^{2}+m_{44}\\ \\ i({\Omega }+m_{45})\\ \par \end {array}}{} \begin {array}{l} 0\\ \\ 0\\ \\ 0\\ \\ -i({\Omega }+m_{45})\\ \\ -{\Omega }^{2}+m_{44}\\ \par \end {array}\right )$where

31 $$\begin{array}{@{}rcl@{}} m_{13} &=& Q_{2}a_{1},\\ m_{23} &=& Q_{2}a_{2},\\ m_{32} &=& 2c_{2}a_{2},\\ m_{31} &=& 2c_{2}a_{1},\\ m_{11} &=& -P_{1}{K_{1}^{2}}+Q_{1},\\ m_{45} &=& -c_{1}KK_{1},\\ m_{33} &=& A-2B\alpha_{0}+ 3W_{g} {\alpha_{0}^{2}}-c_{0}{K_{1}^{2}},\\ m_{43} &=& -Q_{3} b_{1}+ 2Q_{4}\lambda_{0}b_{2}-3\beta {\lambda_{0}^{2}}\gamma b_{2},\\ m_{44} &=& -{w_{0}^{2}}-c_{1}{K_{1}^{2}}-c_{1}K^{2}-{\beta\lambda_{0}^{3}}\gamma+Q_{3}\lambda_{0}+Q_{5}. \end{array} $$

The condition that (30) has a non-trivial solution requires its determinant to be zero. This gives the eigenvalue equation:

32 $$ {\Omega}^{8}-P{\Omega}^{6}-T{\Omega}^{5}+M{\Omega}^{4}+N{\Omega}^{3}+R{\Omega}^{2}+Z{\Omega}+Q = 0. $$

where

33 $$\begin{array}{@{}rcl@{}} P &=& m_{11}^{2}+m_{33}+ 2m_{44}-4{w_{0}^{2}},\\ T &=& 4w_{0}m_{45}.\quad \end{array} $$

34 $$\begin{array}{@{}rcl@{}} N &=& w_{0}(4m_{33}m_{45}-4m_{11}^{2}m_{45}).\quad \end{array} $$

35 $$\begin{array}{@{}rcl@{}} M &=& 2m_{33}m_{44} - 2m_{31}m_{11}m_{13} + m_{11}^{2}(2m_{44} + m_{33} - 4{w_{0}^{2}})\\ && +\, m_{44}^{2} + 4m_{33}{w_{0}^{2}} - m_{45}^{2}. \end{array} $$

36 $$\begin{array}{@{}rcl@{}} R&=&2m_{11}^{2}m_{33}m_{44}+ 4m_{23}m_{44}m_{32}m_{11}-m_{33}m_{44}^{2}-(m_{11}m_{44})^{2}+ 8m_{11}m_{31}m_{13}{w_{0}^{2}}\\ && -\, m_{33}m_{45}^{2} - (m_{11}m_{45})^{2}-4{w_{0}^{2}}m_{11}^{2}m_{33}. \end{array} $$

37 $$ Z=w_{0}m_{45}(8m_{11}m_{13}m_{31}-4m_{11}^{2}m_{33}). $$

38 $$ Q = 2m_{45}^{2}m_{11}m_{13}m_{31}-m_{11}^{2}m_{33}m_{45}^{2}-2m_{11}m_{13}m_{31}m_{44}^{2}+m_{11}^{2}m_{33}m_{45}^{2}. $$

**Fig. 1:**
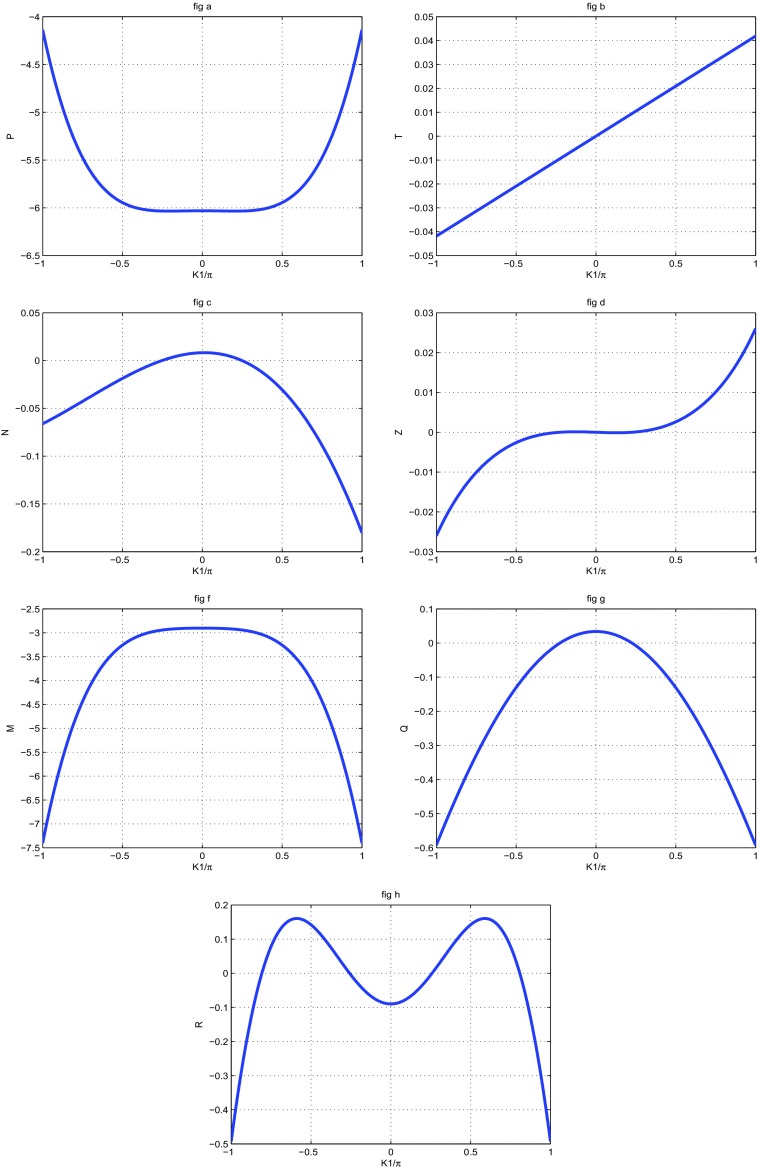
Parameter of the non-linear dispersion relation (41) as function of wave-number*K*_1_ for *m* = 300*amu*, *a* = 4.41*A*^∘^^− 1^, *χ* = 1.2*eV* *A*^∘^^− 1^, *D* = 4.05*eV*, *V* = 0.05*eV*, *k* = 2.04, *ξ* = 2.04, *r* = 0.3*A*^∘^, *I* = 300*amu*, $\varphi _{0}= 10^{-3} A^{\circ }{}^{-1}$

**Fig. 2:**
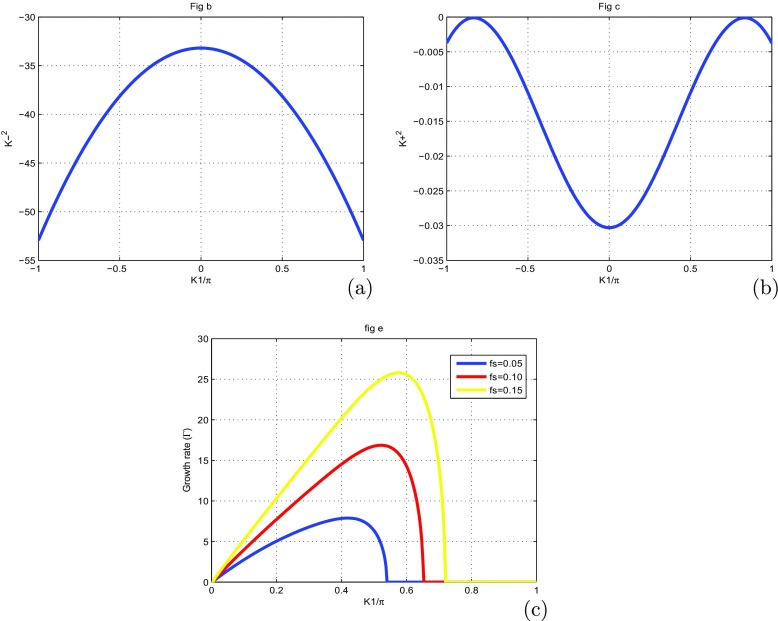
Growth rate and solutions of (27) for *m* = 300*amu*, *a* = 4.41*A*^∘^^− 1^, *χ* = 1.2*eV* *A*^∘^^− 1^, *D* = 4.05*eV*, *V* = 0.05*eV*, *k* = 2.04*eV* *A*^∘^^− 2^, *ξ* = 2.04*eV*, *r* = 0.3*A*^∘^, *I* = 300*amu*, *φ*_0_ = 10^− 3^*A*^∘ − 1^

**Fig. 3:**
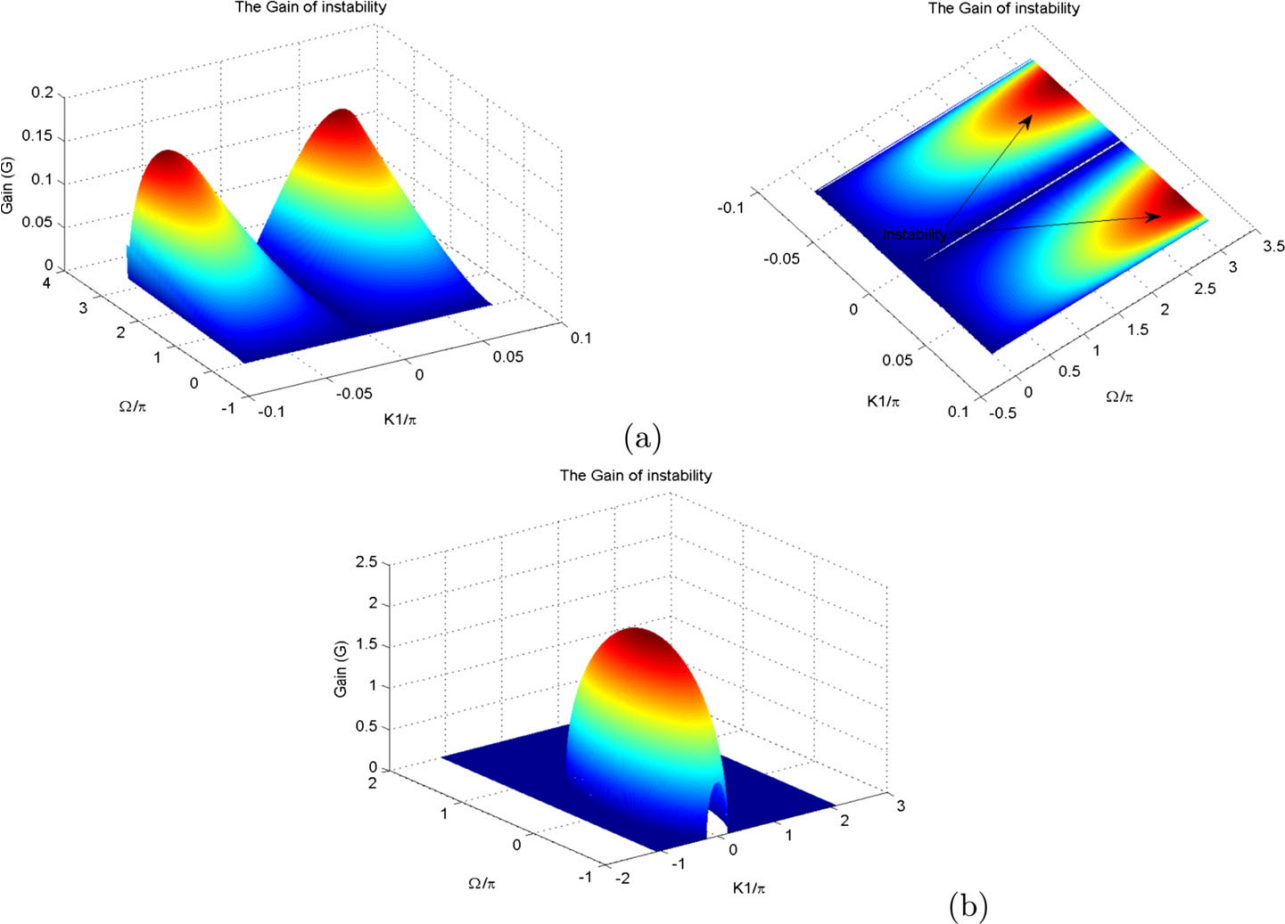
MI integrated gain as a function of *K*1 and Ω: We observe two peaks if *K*1 ∈ [− 45*π*;72*π*], which correspond to the instability domains (see panel Fig. 3a), for *m* = 300*amu*, *a* = 4.41*A*^∘^^− 1^, *χ* = 1.2*eV* *A*^∘^^− 1^, *D* = 4.05*eV*, *ξ* = 2.04*eV*, *V* = 0.05*eV*, *k* = 2.04*eV*, *ξ* = 2.04*eV*, *r* = 0.3*A*^∘^, *I* = 300*amu*, *l*_*s*_ = 1 and *f*_*s*_ = 1. We noted that the two peaks become one common gain peak if *K*1 increases (see panel Fig. 3b). This explains the phenomenon of MI in the system under our study

**Fig. 4:**
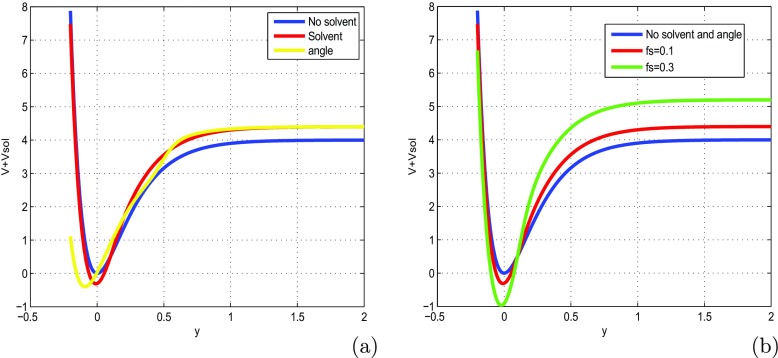
Sum of Morse and solvent potentials when *g*(*ϕ*_*j*_, *𝜃*_*j*_)=$r(\cos \phi _{j}+\cos \theta _{j})$ is constant where *m* = 300*amu*, *a* = 4.5*A*^∘^^− 1^, *D* = 4.04*eV*, *r* = 01.3*A*^∘^, *I* = 300*amu*, *𝜃* = *pi*/4*rd*, *ϕ* = *pi*/5*rd* (**a**) *f*_*s*_ = 2 (**b**)

To make sure that our system is stable, we have plotted the coefficients of (32) in Fig. 1, as function of wave number *K*1. We also plot the solution of the (12) (see Fig. 2a, b) and the growth rate of instability (see Fig. 2c). The MI gain *G*(Ω, *K*_1_)=|*Im*(*K*_1_(Ω))| is the largest value among those corresponding to the various branches of the dispersion relation. The full spectrum of the MI gain *G*(Ω, *K*_1_) was found from a numerical solution of the dispersion (32). In Fig. 3, the corresponding gain is shown in tridimensional and surface plots against the wavenumber *K*_1_ and the frequency of perturbation Ω. This is a first confirmation of the possibility of MI in the system under our study. The major remark is that the dispersion coefficient’s relation curve which oscillating motion. This behavior is due to the introduction of the torsional movement and the solvent interaction factor. In context of charges transport, the charges are confined in some specific sites under the influence of the oscillation of the coefficients of (32). The charges can jump from one atom to another. The rotational movement can prevent the spreading of charges. Taking into account the solvent factor in the DNA model, the energy can be trapped within the molecule, and consequently the charges are stored in a site. There is no longer propagation or transfer of genetic information. The gene that encoded the information may not copy properly. This explains some chromosomal diseases. We have plotted in Fig. 2c the behavior of the growth rate of instability according to the solvent interaction factor *f*_*s*_. When it is high, the peak of instability increases. The solvent factor clearly fluctuates with instability. The solvent factor amplifies the instability gain and increases the domains of instability within the molecule (see in Fig. 2c). Therefore, in the presence of environment ions and the solvent molecules, DNA tries to minimize its energy by altering its conformation. This in turn changes the charge transport properties through DNA. Effectively, the interaction of sugar group with polar water molecules and ions changes the electron cloud at the sugar group, which in turn can change the electron cloud at the base. This result was suggested by Voityuk et al. [35]. Certain parameters used in the present work have been borrowed in the references [22, 27, 31] and others have been modified by the authors. We can see that: *l*_*s*_ and *f*_*s*_ tunes the width of the solvent barrier. In fact, the potential *U*_*sol*_ introduces a potential barrier in the Morse potential, materialized by the factor *f*_*s*_*D*, as shown in Fig. 4. The potential barrier can prevent unstacked bases re-closing when the H-bonds have been broken. In this case, lattice vibrations under charge dynamics will remain well inside the Morse potential and will be prevented from jumping the barrier. The charge can be trapped by the barrier and there is no longer propagation of charges within the molecule. We can conclude that the water and the ions present in the hydration of the DNA can affect the transport of charges. In fact, when *λ*_*j*_ < *l*_*s*_, the hydrogen bonds are broken and linked to solvent molecules. The plateau that appears for *λ*_*j*_ > *l*_*s*_ represents a situation where the unstacked regions are stabilized by free solvent, so that they can no longer move in any direction (Fig. 4).

## Numerical analysis of MI and DNA wave patterns

**Fig. 5:**
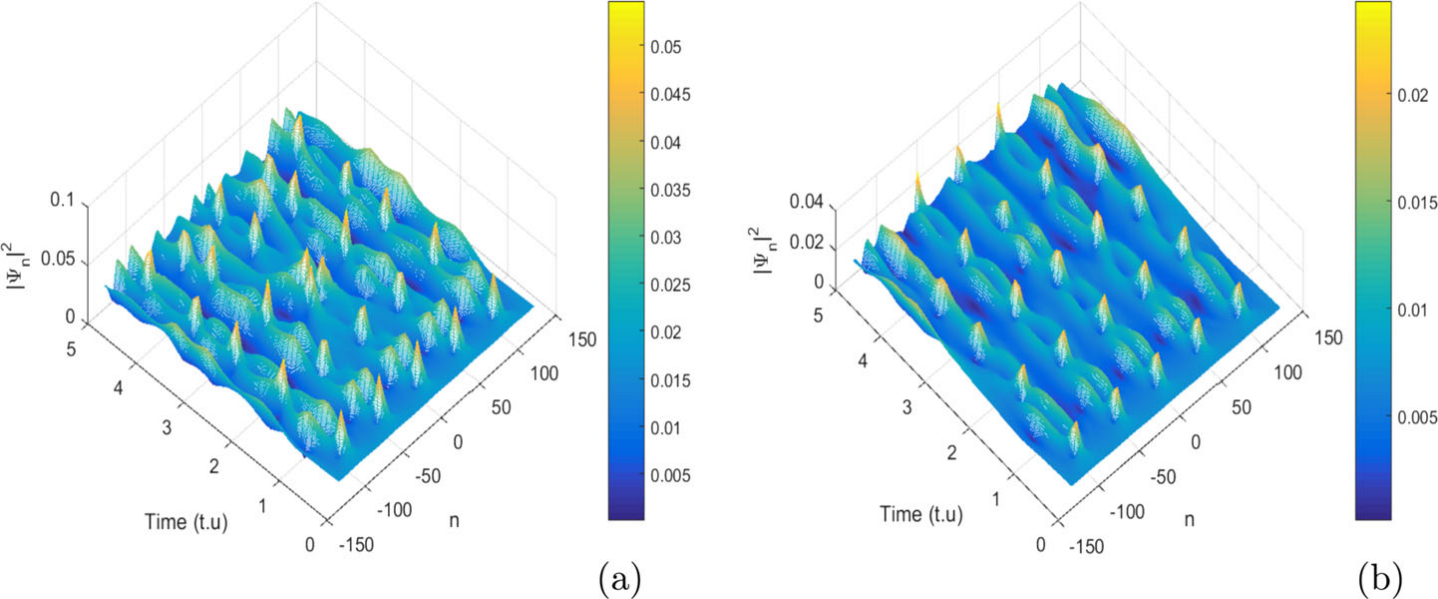
The manifestations of modulational instability of charge transport in the DNA model under the influence of charge-vibrational and rotational coupling constant without solvent interaction. The main remark is that the emergence of the charges exists. These are propagated within the molecule. The numerical density of these charges decreases in our model with the coupling factor. This was demonstrated in our previous work [33]. The charges can migrate in all the directions of the wave propagation. The charges can occupy the pairs of bases or can be trapped by pairs bases. This explains the existence of small radiations at certain sites of the molecule. The constant values used are as follows: *m* = 300*amu*, *a* = 4.4*A*^∘^^− 1^, *D* = 4.1*eV*, *V* = 0.1*eV*, *r* = 0.3*A*^∘^, *I* = 300*amu*, *m* = 300*amu*, with **a** *χ* = 0.6*eV* *A*^∘^^− 1^ and **b** *χ* = 0.8*eV* *A*^∘^^− 1^

**Fig. 6:**
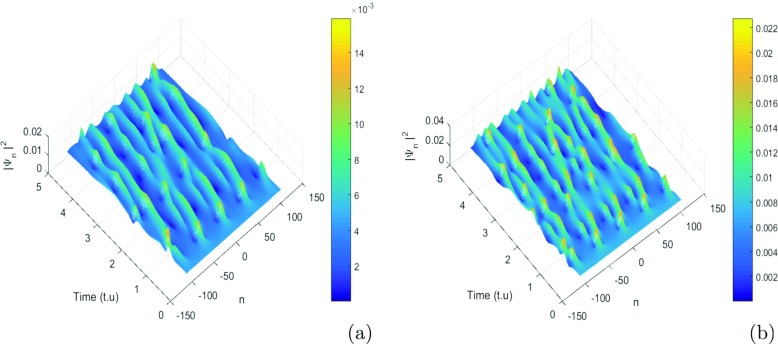
Localized structures with solvent interaction. Taking into account the interaction factor of the solvent, the plane wave solutions should break in trains of localized structures. However, we note a new result when increasing the interaction factor of the solvent *f*_*s*_—the localized structures also increase (see Fig. 6a, b). We can conclude that the increase in the solvent factor must saturate the panel network with localized structures. Therefore, the charge transfer can be interrupted by the solvent. So we find ourselves in a competition between the transport and the capture of the load by the solvent. The ions present in the hydration of the DNA can affect the transport of the charges because the solvent can induce the ionization of the double helices of the DNA. The genetic information within the molecule is thus disturbed. The vital phenomena that exist in the molecule, namely transcription, translation, and even reproduction, can be damaged. This may explain some chromosomal mutations. The values of parameters are *m* = 300*amu*, *a* = 4.41*A*^∘^^− 1^, fixing *l*_*s*_ = 2*A*^∘^ and *χ* = 1.2*eV* *A*^∘^^− 1^, *I* = 300*amu*, *D* = 4.1*eV*, *V* = 0.05*eV*, *ξ* = 2.04*eV*, *k* = 2.04 and *r* = 2.3*A*^∘^ *f*_*s*_ = 0.5 see Fig. 6a and *f*_*s*_ = 0.70 see Fig. 6b

**Fig. 7:**
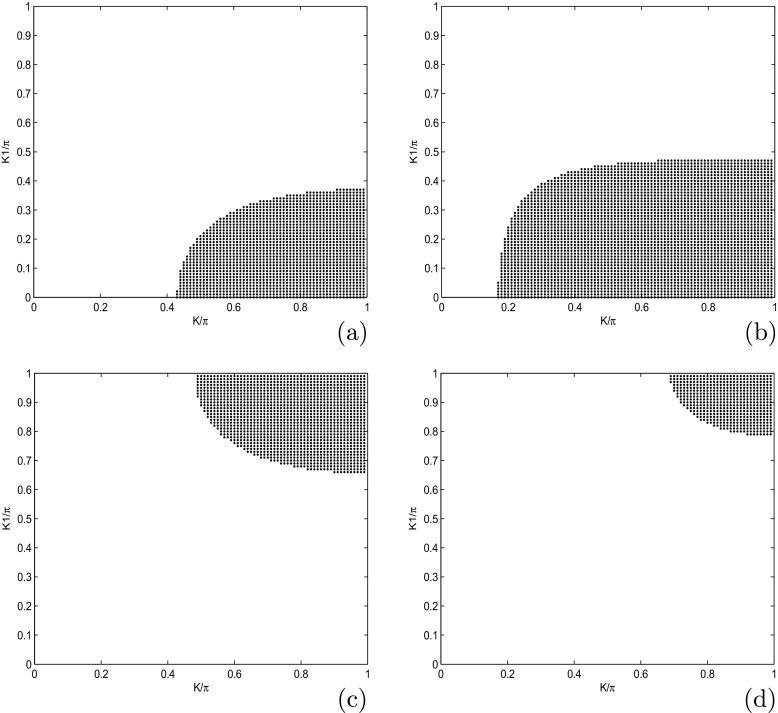
Diagrams of stability/instability. *Black domains* in stability/instability diagrams are regions of instability, the plane wave solutions are expected to break into trains of solitons (localized structures). The other regions (*white domains*) are where the propagation of waves are expected to remain stable under the modulation. We have fixed *l*_*s*_ = 1 and taking *f*_*s*_ = 0.2 and *f*_*s*_ = 0.5, one obtains, respectively, Fig. 7a and b. In general, increasing *f*_*s*_ and fixing *l*_*s*_, obviously increases instability domains. Fixing *f*_*s*_ = 1 and taking *l*_*s*_ = 0.02 and *l*_*s*_ = 0.05, one obtain the Fig. 7c and d. We note that increasing *l*_*s*_ and fixing *f*_*s*_, obviously increases the stability domains, i.e., reduces instability domains. The stability/instability dramatically modified by the solvent factor, both quantitatively and qualitatively (enlarged unstable wavenumber region). Other parameters are *m* = 300*amu*, *a* = 4.41*A*^∘^^− 1^, *χ* = 1.2*eV* *A*^∘^^− 1^, *D* = 4.1*eV*, *V* = 0.05*eV*, *k* = 0.04*eV* = *ξ*, *r* = 0.3*A*^∘^ and *I* = 300*amu*

**Fig. 8:**
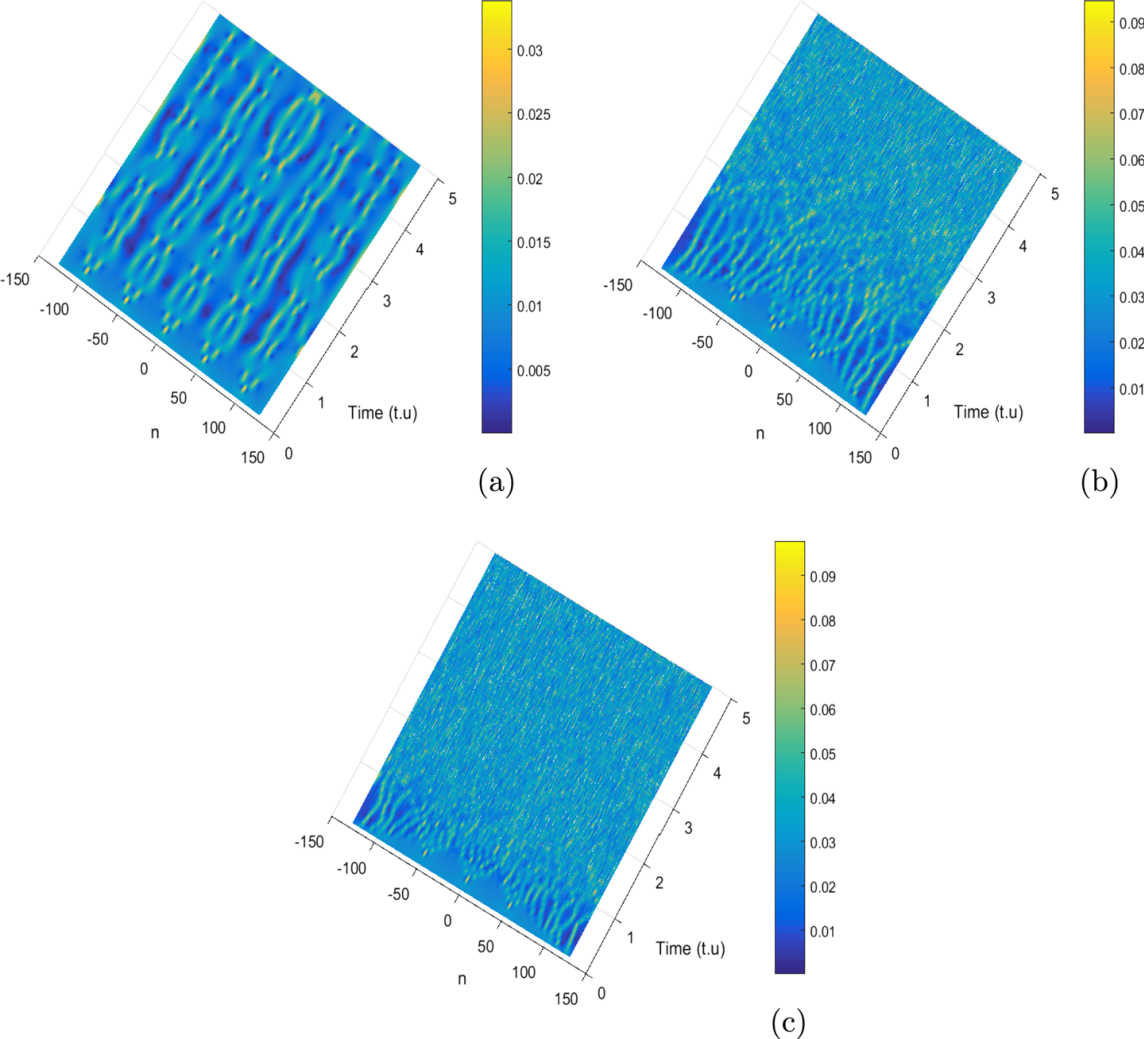
Localized structures with simultaneous effect of coupling constant and solvent interaction for *m* = 300*amu*, *a* = 4.41*A*^∘^^− 1^, fixing *l*_*s*_ = 2*A*^∘^, *I* = 300*amu*, *D* = 4.0*eV*, *V* = 0.005*eV*, *ξ* = 2.04*eV*, *k* = 02.04*eV*, *f*_*s*_ = 3, and *r* = 2.3*A*^∘^, with *f*_*s*_ = 0.4 and *χ* = 1.2*eV* *A*^∘^^− 1^ see Fig. 8a, *f*_*s*_ = 0.4 and *χ* = 0.4*eV* *A*^∘^^− 1^ (see Fig. 8b and *f*_*s*_ = 0.8 and *χ* = 0.75*eV* *A*^∘^^− 1^ see Fig. 8c). We note that in the presence of the solvent factor and the coupling constant, the density of charge can migrate in all the directions of the wave propagation (see Fig. 8a), but from Fig. 8b, we observe that the density of charges finally vanishes in small radiations when the coupling constant *χ* decreases. This result was suggested by Tabi et al. [34], which considered the DNA model in the presence of thermal effect. By increasing *f*_*s*_ and *χ*, the propagation of charges is not possible or not perceptible (see Fig. 8c). In fact, after the H-bonds are broken due to the water, the bases are maintained opened. Therefore many chemical reactions can occur within the molecule. Consequently, the DNA molecule opens locally and exposes the base pairs at the surface. In this case, the migration of charges is not perceptible. The localization and the propagation of charges in our model can become chaotic and beyond our control when *f*_*s*_ and *χ* increases. Consequently, the information within the molecule and the DNA functions can be blocked, see Fig. 8c

In order to check the validity of our analytical approach and to explore the formation of localized modes with solvent interactions, we exactly solve the set of nonlinear coupled differential equations by using the standard Fourier transform method, with an integration time step of 0.055*s*. In our numerical simulations, the initial conditions at time *t* = 0, are coherently modulated plane wave of the form:

39 $$\begin{array}{@{}rcl@{}} \lambda_{n}&=&\lambda_{0}(1 + 0.01\cos(Kn))\cos(K_{0}n),\\ \varphi_{n}&=&\varphi_{0}(1 + 0.01\exp(i Kn))\exp(i K_{0}n), \\ \psi_{n}&=&\psi_{0}(1 + 0.01\exp(i Kn))\exp(i K_{0}n). \end{array} $$

where *λ*_0_ = 0.0035 and *ψ*_0_ = *φ*_0_ = 0.002 are the initial displacement and the initial probabilities for the charge transport. For our study, we use *K* = 0.71*π* and *K*_0_ = 0.55*π* that are values are the wavenumbers for the perturbation and the carrier waves. These values correspond to a point belonging to the red region of the instability of Fig. 3. We use these values of parameters, and we investigate the emergence of localized structures in our model. Since a biological molecule like DNA is always in an environment (or heat bath) with finite temperature, the state and properties of the soliton are affected by the temperature and the medium. For this point of view, Tabi et al. [17, 18] and Mvogo et al. [32] are already showed that, the perturbations of thermal noise do not destroy localized structures but contributes to enhancing and ensuring better transport of energy into the molecule. Among the biomolecules that are likely to be used as electrical wire, those of DNA have many advantages. They have the faculty of assembling spontaneously in ordered assemblies (self-assembly), of duplicating themselves, and of adopting varied conformations. The electrons that gravitate around the atoms on the farthest orbits are very little constrained to remain in this orbit. This idea also justifies the peak of charges observed in charges patterns. The charge can be jumped from one atom to another after long times localization in specific sites because the charges are inside the potential of interaction of solvent. However, from a theoretical point of view, the solvent interaction plays an important role in its internal dynamics. So it is necessary to explore the role of solvent in the process of formation of localized structures. As is shown in Fig. 5, the initial condition tends to disintegrate during the propagation, leading to a break-up of the wave into a pattern of wave trains. This result confirms also our analytical predictions. We observe that the patterns do not saturate and their amplitude decreases with decreasing time. The efficiency of transport of charges in the study is affected by stretching of the molecule due to the solvent. Taking into account the solvent factor, the energy is trapped within the molecule, and consequently the charges are stored in a specific site for efficiency (see Fig. 5a and b). There is no longer propagation or transfer of genetic information. The gene that encoded the information may not copy properly. In fact, the ions under the effect of water can capture one, two, or three electrons and is transformed into a more oxidizing molecule. The presence of a single electron gives these molecules most of the time great instability and a high reactivity. In this case, electrical charges circulate and induce energy reactions. They bring about rapprochements, selective distances in time, and space. They modify the energy state of atoms, a state that is transmitted to other atoms, which induces electromagnetic emissions. Consequently, the transmission of information between the cells by the electrons and the photons is perturbed. The solvent factor *l*_*s*_ or *f*_*s*_ can also lead to the loss of DNA fragments and thus, during the repair process, the pairing of non-homologous chromosomes (which do not belong to the same pair) leading to the loss or amplification of the chromosomes. We also note that the charge patterns are sensitive to solvent interactions, as they get more localized with the increasing of *l*_*s*_ (see Fig. 6). We also note that the density of charges is highly localized when the time increases, as is shown in Fig. 6b, where the red regions correspond to highest the concentration of charges. In this case, the charges are stored in specific domains and the vital process like duplication of DNA molecule can be stopped (Fig. 7). Further increasing simultaneously *f*_*s*_ and *l*_*s*_, has revealed another interesting feature, as patterns of charges can be localized over a long time for the highest values of *f*_*s*_ and *l*_*s*_. We also point out the role of coupling constant and solvent interaction in the second case; we observe that the localized structures tend to disappear (see Fig. 8b, and c). In general, the effect of solvent dampens out the amplitude soliton in the wave patterns and tends to form peaks that seem to enhance the information in the molecule. We note that the localized structures or excitations disappear. In fact, the softness of the biomolecule can also indirectly cause damage to cells by creating free radicals. Free radicals are extremely reactive molecules due to the presence of free electrons (ions), created by the separation of water molecules. They can form compounds such as hydrogen peroxide or superoxide, which can induce chemical reactions within cells. As a result of these chemical changes, the cells can undergo various structural changes that lead to their death or transform their function. In this case, we have the non-localization and the transfer of loads in the molecule. Then, the charges are mapped to the atomic charges of system. The spreading of loads vanishes and the efficiency drops steeply with the increasing of coupling *χ* and *f*_*s*_ see patterns of Fig. 8.

## Conclusions

We discuss solvent interactions and the possibility of generation of soliton-like excitations along the DNA model, based on a set of coupled nonlinear equations. The linear stability has been studied under the continuum approximation and the emergence of localized structures in DNA model have been displayed. From numerical methods, we have plotted the region of stability/instability due to the increase of solvent interaction. We also showed that our model could be subjected to MI, as indicated by the numerical representation of the growth rate of instability. Our analytical predictions have been verified numerically, where the pattern of charges has been displayed. In this case, increasing the solvent factor, the domains of instability increases and prevents charge spreading. The potential barrier brought by the term *f*_*s*_*D* destroys the H-bonds and blocks charge spreading. We also show that the solvent factor does not completely destroy the emergence of localized modes but prevents the propagation of the flow of information in the cells. We note that the charge patterns are sensitive to solvent interactions as they get more localized with increasing *f*_*s*_ and *l*_*s*_. Further increasing *f*_*s*_, has revealed another interesting feature, as patterns of charge can be localized over a long time for highest values of *l*_*s*_. The solvent molecules can collide and bring additional charges in the patterns. This explains the highest density of charges observed in Fig. 6. Taking into account the solvent factor in our model, the localized structures become robust with high values of *l*_*s*_. The spectrum of behaviors is displayed in Fig. 6. We can see that, the localized modes persists with robustness behavior a much better candidate of non-linear modes responsible for a locally open state where biological functioning takes place. The efficiency of transport of charges is affected by stretching of the molecule. The density of loads vanishes and the efficiency drops steeply. Thereafter, the life-time of charges decreases with an increase of *f*_*s*_ reinforces the spatial confinement of the charge carrier in specific domains. Increasing *f*_*s*_ and *l*_*s*_ in the model also revealed that due to the environment effect of the charges, we observe that the bubbles form spatiotemporal “hot-spots”, which inhibit charge propagation along the strands and enhance its confinement. Thereafter, the flux of charges is concentrated for a long times in specific sites. Consequently, the transfer of information along the molecule is also blocked. This explains some chromosome diseases or mutations due to an accumulation of mutations that increase its proliferation capabilities, the instability of its genome, and its ability to escape systems that eliminate abnormally proliferating cells. Our results obtained suggest that it is possible to reduce some chromosome diseases by including the solvent factor interactions and *χ* in DNA model. Taking into account the effect of solvent factor and the coupling constant, the density of charges can vanish, and only the small quantity of charges can migrate in the DNA model. We can conclude that, the solvent factor can be facilitators or inhibitors charge transport in the modified DNA model and the good conduction of current is also depend on the flux of charges carrier. However, theoretical investigations of charge transport and localization in DNA are complicated, not only due to the intrinsic disorder caused by the different nucleotides and dynamics of bases present in DNA but also because of the softness of the biomolecule. However, in this present study, other factors are not considered, such as viscosity and diffusion effect. It is important to consider their influence in the charge transport model, which is work that we are currently doing.

## Appendix

The total Hamiltonian of our system is:

40 $$ H=H_{1}+H_{2}+H_{3} $$

Now, we use semiclassical equations of motion [22, 23], i.e., we treat the quantum charge mechanically and the vibrational and rotational motion classically. The equation for the time evolution of the charge probability amplitude is obtained with the use of Schrödinger equation: $i\hbar \frac {d|\varphi _{c}(t)>}{dt}=(H_{2}+H_{3})|\varphi _{c}(t)>$ where *H*_2_ and *H*_**3**_ are the charge and the coupling Hamiltonian, respectively i.e.,

41 $$ i\hbar \frac{d}{dt}\varphi_{j} = -V\left( {\varphi_{j + 1} + \varphi_{j - 1} } \right) + \chi \lambda_{j} \varphi_{j}. $$

where*φ*_*j*_ is the probability amplitude for the charge carrier located at the *jth* base pair. To obtain our equations of motion, we use the Newton equations: $\frac {d \lambda _{j}}{dt}=\frac {\partial H}{\partial p_{j}}$, $\frac {d p_{j}}{dt}=-\frac {\partial H}{\partial p_{j}}$ and $p_{j} = m\frac {d \lambda _{j}}{d t}$. Recall:

42 $$ \frac{d\tanh(x)}{dx}=\frac{1}{cosh^{2}(x)}=\sec\!\text{h}^{2}(x), $$

we have

43 $$ \frac{dV_{sol}}{d\lambda_{j}}=\frac{\sqrt2D_{j}f_{s}}{l_{s}}\sec\!\text{h}^{2}\left( \frac{\sqrt{2}\lambda_{j}-g(\phi_{j},\theta_{j})}{l_{s}}\right). $$

44 $$ \frac{dV_{sol}}{d\phi_{j}}=-\frac{r\sin\phi_{j}D_{j}f_{s}}{l_{s}}\sec\!\text{h}^{2}\left( \frac{\sqrt{2}\lambda_{j}-g(\phi_{j},\theta_{j})}{l_{s}}\right). $$

45 $$ \frac{dV_{sol}}{d\theta_{j}}=-\frac{r\sin\theta_{j}D_{j}f_{s}}{l_{s}}\sec\!\text{h}^{2}\left( \frac{\sqrt{2}\lambda_{j}-g(\phi_{j},\theta_{j})}{l_{s}}\right). $$

After using the above equation, the Newton equations of motion for the stretching *w*_*j*_**,***λ*_*j*_, and the rotational *𝜃*_*j*_, *ϕ*_*j*_ motions becomes:

46 $$ m{ \ddot{w}_{j}}=k(w_{j + 1}+w_{j-1}-2w_{j}). $$

47 $$\begin{array}{@{}rcl@{}} m\ddot{\lambda_{j}}&=&k(\lambda_{j + 1}+\lambda_{j-1}-2\lambda_{j})+ 2\sqrt{2}a D(\exp(-a\sqrt{2}\lambda_{j}-g(\theta_{j},\phi_{j}))-1)\\ && \times\, (\exp(-a\sqrt{2}\lambda_{j}-g(\theta_{j},\phi_{j})))-\chi\lambda_{j}|\varphi_{j}|^{2}\\ && +\, \frac{\sqrt{2}D_{j} f_{s}}{l_{s}}\sec\!\text{h}^{2}\left( \frac{\sqrt{2}\lambda_{j}-g(\phi_{j},\theta_{j})}{l_{s}}\right). \end{array} $$

48 $$\begin{array}{@{}rcl@{}} I\ddot{\phi_{j}}&=&\xi(\phi_{j + 1}+\phi_{j-1}-2\phi_{j})-2raD\sin\phi_{j}(\exp(-a\sqrt{2}\lambda_{j}-g(\theta_{j},\phi_{j}))-1)\\ && \times\, (\exp(-a\sqrt{2}\lambda_{j}-g(\theta_{j},\phi_{j})))\\ && +\, \frac{D_{j} r f_{s} \sin \phi_{j} }{l_{s}}\sec\!\text{h}^{2}\left( \frac{\sqrt{2}\lambda_{j}-g(\phi_{j},\theta_{j})}{l_{s}}\right). \end{array} $$

49 $$\begin{array}{@{}rcl@{}} I\ddot{\theta_{j}}&=&\xi(\theta_{j + 1}+\theta_{j-1}-2\theta_{j})-2raD\sin\theta_{j}(\exp(-a\sqrt{2}\lambda_{j}-g(\theta_{j},\phi_{j}))-1)\\ && \times\, (\exp(-a\sqrt{2}\lambda_{j}-g(\theta_{j},\phi_{j})))\\ && +\, \frac{ D_{j} r f_{s} \sin \theta_{j}}{l_{s}}\sec\!\text{h}^{2}\left( \frac{\sqrt{2}\lambda_{j}-g(\phi_{j},\theta_{j})}{l_{s}}\right). \end{array} $$

Let:

50 $$\begin{array}{@{}rcl@{}} X_{j}=\frac{\theta_{j}+\phi_{j}}{\sqrt{2}}, \\ {\Psi}_{j}=\frac{\theta_{j}-\phi_{j}}{\sqrt{2}}, \end{array} $$

51 $$\begin{array}{@{}rcl@{}} x_{j}=\frac{X_{j}}{\sqrt{2}},\\ \psi_{j}=\frac{{\Psi}_{j}}{\sqrt{2}}, \end{array} $$

Our priority is to transform (48) and (49) into one equation. Substituting (51) and (52) into equations 48-49, we obtain the following equation:

52 $$\begin{array}{@{}rcl@{}} I\ddot{\psi_{j}}&=&\xi(\psi_{j + 1}+\psi_{j-1}-2\psi_{j})-2raD_{j}\sin\psi_{j}\cos x_{j}(\exp(-a\sqrt{2}\lambda_{j}-g(x_{j},\psi_{j}))-1)\\ && \times\, (\exp(-a\sqrt{2}\lambda_{j}-g(x_{j},\psi_{j})))\\ && +\,\frac{ D_{j} r f_{s} \cos x_{j} \sin\psi_{j}}{l_{s}}\sec\!\text{h}^{2}\left( \frac{\sqrt{2}\lambda_{j}-g(x_{j},\psi_{j})}{l_{s}}\right). \end{array} $$

where $g(X_{j},{\Psi }_{j})=g(x_{j},\psi _{j})= 2r(\cos x_{j}\cos \psi _{j})$. Equation (46) has plane solutions. Our attention will be focused on the nonlinear (47–49). For small angular motion we have $\cos x_{j}\simeq 1$, $\sin \psi _{j}\simeq \psi _{j}$ and $\exp (-a\sqrt {2}\lambda _{j}-g(x_{j},\psi _{j}))\simeq \exp (-a\sqrt {2}\lambda _{j})$ because $g(x_{j},\psi _{j})\simeq 2r$ and *r* is the constant.

Using this approximation, and (52) becomes:

53 $$\begin{array}{@{}rcl@{}} I\ddot{\psi_{j}}&=&\xi(\psi_{j + 1}+\psi_{j-1}-2\psi_{j})-2raD_{j}\psi_{j}(\exp(-a\sqrt{2}\lambda_{j})-1) (\exp(-a\sqrt{2}\lambda_{j}))\\ &&+\,\frac{ 2 D_{j} r f_{s} \psi_{j}}{l_{s}}\sec\!\text{h}^{2}\left( \frac{\sqrt{2}\lambda_{j}-2r}{l_{s}}\right). \end{array} $$

Finally, after using all cited approximations, the equations of motion become three discrete equations for three variables,

54 $$ i\hbar \frac{d}{dt}\varphi_{j} = -V\left( {\varphi_{j + 1} + \varphi_{j - 1} } \right) + \chi \lambda_{j} \varphi_{j}. $$

55 $$\begin{array}{@{}rcl@{}} m\ddot{\lambda_{j}}\!&=&\!k(\lambda_{j + 1} \,+\, \lambda_{j-1} \,-\, 2\lambda_{j}) \,+\, 2\sqrt{2}a D_{j}(\exp(-a\sqrt{2}\lambda_{j})\,-\,1) (\exp(-a\sqrt{2}\lambda_{j})) \,-\, \chi\lambda_{j}|\varphi_{j}|^{2} \\ && +\,\frac{\sqrt{2}D_{j} f_{s}}{l_{s}}\sec\!\text{h}^{2}\left( \frac{\sqrt{2}\lambda_{j}-2r}{l_{s}}\right). \end{array} $$

56 $$\begin{array}{@{}rcl@{}} I\ddot{\psi_{j}}&=&\xi(\psi_{j + 1}+\psi_{j-1}-2\psi_{j})-2raD_{j}\psi_{j}(\exp(-a\sqrt{2}\lambda_{j})-1) (\exp(-a\sqrt{2}\lambda_{j}))\\ && +\, \frac{2 D_{j} r f_{s} \psi_{j}}{l_{s}}\sec\!\text{h}^{2}\left( \frac{\sqrt{2}\lambda_{j}-2r}{l_{s}}\right). \end{array} $$

Let $ \sec \!\text {h}^{2} (y)= 1-y^{2}$. After expanding the terms in exponential up to the third order and using the continuum approximation, we obtain the following equations

57 $$ i\frac{d}{dt}\varphi= P_{1}\frac{\partial^{2}\varphi}{\partial x^{2}}+Q_{1}\varphi+Q_{2}\lambda\varphi. $$

58 $$ \ddot{\lambda}+c_{0}\frac{\partial^{2}\lambda} {\partial x^{2}}+A\lambda+B\lambda^{2}+W_{g}\lambda^{3}+c_{2}|\varphi|^{2}= 0. $$

59 $$ \ddot{\psi}+c_{1}\frac{\partial^{2}\psi} {\partial x^{2}}+Q_{3}\psi\lambda+Q_{4}\psi\lambda^{2}-\gamma\beta\psi\lambda^{3}+Q_{5}\psi= 0. $$

where $W_{g}=\frac {4a^{2}D_{j}}{m}$, $\alpha _{0}=\frac {3a\sqrt {2}}{2}$, $c_{0}=\frac {-k}{m}$, $c_{1}=\frac {-\xi }{I}$, $P_{1}=-\frac {2V}{\hbar }$, $Q_{1}=-\frac {V}{\hbar }$, $Q_{2}=\frac {\chi }{\hbar }$, $\gamma =\frac {7a^{2}}{3}$, $\beta =\frac {m r W_{g}\sqrt {2}}{2I}$, $A=\frac {8 r D_{j} f_{s}}{{l_{s}^{3}}}+W_{g}$, $B=\alpha _{0} W_{g}+ \frac {2 \sqrt {2} D_{j} f_{s}}{ {l_{s}^{3}}}$, $Q_{3}=\frac {8 \sqrt {2}r^{2} D_{j} f_{s}}{{l_{s}^{3}}}-\beta $, $Q_{4}=-\frac {4 r D_{j} f_{s}}{ {l_{s}^{3}}}+\beta \alpha _{0}$ and $Q_{5}=\frac {2 r D_{j} f_{s}}{m l_{s}}\left (1-\frac {4 r^{2}}{{l_{s}^{2}}}\right )$.

## References

[1] Ly D, Sanii L, Schuster GB (1999). Mechanism of charge transport in DNA: internally-linked anthraquininone conjugates support phonon–phononassisted polaron hopping. J. Am. Chem. Soc..

[2] Bixon M, Jortner J (2000). Energetic control and kinetics of hole migration in DNA. J. Phys. Chem. B.

[3] Nunez M, Hall DB, Barton JK (1999). Long-range oxidative damage to DNA: effects of distance and sequence. Chem-Biol.

[4] Wynveen A, Kornyshev (2004). Nonlinear effects in the torsional adjustment of interacting DNA. Phy. Rev. E.

[5] Hwang S-Y (2010). Charge transport in DNA with a base pair opening. J. Korean Phys. Soc..

[6] Hennig, D.: Solitonic energy transfer in a coupled exciton–vibron system. Phys. Rev. E, **61** (2000) 10.1103/physreve.61.4550 11088255

[7] Toko D, Woulaché L, Tabi CB, Kavitha L, Mohamadou A, Kofané TC (2013). Breather-like solutions of the twisted DNA with solvent interaction. Phys. Chem. Biophys.

[8] Dauxois T, Peyrard M, Bishop AR (1993). Entropy-driven DNA denaturation. Phys. Rev. E..

[9] Peyrard M, Bishop AR (1989). Statistical mechanics of non linear model for DNA denaturation. Phys. Rev. Lett..

[10] Zdravkovic S, Sataric M (2008). Nonlinear Schrdinger equation and DNA dynamics. Phys. Lett. A.

[11] Tabi CB, Mohamadou A, Kofané TC (2008). Soliton-like excitation in a non linear model of DNA dynamics with viscosity. Math. Biosci. Eng..

[12] Barbi M, Cocco S, Peyrard M (1999). Helicoidal model for DNA opening. Phys. Lett. A.

[13] Cocco S, Monasson R (1999). Statistical dynamics of torque induced denaturation of DNA. Phys. Rev. Lett..

[14] Yomosa S (1984). Solitary excitations in deoxyribonucleic acid (DNA) double helices. Phys. Rev. A.

[15] Arévalo E, Gaididei Y, Mertens FG (2002). Solitons dynamics in damped and forced Boussinesq equation. Eur. Phys. J. B.

[16] Arévalo E, Gaididei Y, Mertens FG, Bishop AR (2002). Thermal diffusion of supersonic solitons in an anharmonic chain of atoms. Phys. Rev. E.

[17] Ekobena Fouda H, Tabi CB, Mohamadou A, Kofane TC (2000). Modulational instability and patterns formation in DNA dynamics. J. Phys. Condens. Matter..

[18] Kalosakas G (2016). Charge transport in DNA: dependence of diffusion coefficient on temperature and electron-phonon coupling constant. Phys. Rev. E.

[19] Zamora-Sillero E, Shapovalov AV, Esteban FJ (2007). Formation, control and dynamics of N localized structures in the Peyrard–Bishop model. Phys. Rev. E.

[20] Fialko NS, Laklno VD (2000). Non linear dynamics of excitations in DNA. Phys. Lett. A.

[21] Fialko NS, Laklno VD (2002). Long-range charge transport in DNA. Regular Chaotic Dyn..

[22] Kaloskas G, Aubry S, Tsironis GP (1998). Polaron solutions and normal-model analysis in the semi classical Holstein model. Phys. Rev. B.

[23] Su WP, Schrieffer JR, Heeger AJ (1980). Solitons excitations in polyacetylene. Phys. Rev. B.

[24] Cherstvy AG, Kornyshev AA, Leikin S (2004). Torsional deformations of double helix in interaction and aggregation. J. Phys. Chem. B.

[25] W Hidayat, A., Sulaiman, S., Viridi, F.: Zen: The viscous and external forces effect on the thermal denaturation of the Peyrard–Bishop model. Phys. Chem. Biophys., 5 (2015)

[26] Silva RAS, Drigo Filho E, Ruggiero JR (2008). A model coupling vibrational and rotational motion the DNA molecule. J. Biol. Phys..

[27] Tabi CB, Mohamadou A, Kofané TC (2008). Soliton excitations in the DNA double helix. Phys. Scr..

[28] Zoli M (2011). Thermodynamics of twisted DNA with solvent interaction. J. Chem. Phys..

[29] Drukker K, Wu G, Schatz G (2001). Model simulations of DNA denaturation dynamics. J. Chem. Phys..

[30] Weber G (2006). Sharp DNA denaturation due to solvent interaction. Europhys. Lett..

[31] Tabi CB, Mohamadou A, Kofané TC (2009). Modulational instability of charge transport in Peyrard–Bishop–Holstein. J. Phys. Condens. Matter.

[32] Mvogo A, Kofané TC, Ben-Bolie G.H. (2014). Discrete energy transport in collagen molecules. Chin. Phys. B..

[33] Ngoubi H, Ben-Bolie GH, Kofané TC (2017). Charge transport in DNA model with vibrational and rotational coupling motions. J. Biol Phys..

[34] Tabi CB, Dang Koko A, Oumarou Doko R, Ekobena HP, Kofané TC (2016). Modulated charge patterns and noise effect in a twisted DNA model with solvent interaction. Physica A.

[35] Voityuk AA (2005). Charge transfer in DNA: hole charge is confined to a single base pair due to solvation effects. J. Chem. Phys..

